# Flexible microneedle array with AuNPs/MXene for visualized electrochemical monitoring of osteosarcoma miR-181b

**DOI:** 10.3389/fbioe.2026.1918744

**Published:** 2026-08-10

**Authors:** Zhong-Sheng Zhu, Hui Zhang, Changchun Lu, Li Du, Shuai Lu, Xin-Qi Cao, Ai Zhong Ding, Nuo Yin

**Affiliations:** Shanghai University of Medicine & Health Science Affiliated Sixth People’s Hospital South Campus, Shanghai, China

**Keywords:** AuNPs-MXene, electrochemical biosensor, microneedle, miR-181b, osteosarcoma

## Abstract

Osteosarcoma monitoring lacks highly sensitive and portable molecular detection methods, creating an urgent clinical need. This study develops a highly sensitive and portable biosensing platform (M-Needle-AuNPs-MXene-EC) integrating flexible microneedle (M-Needle) sampling, gold nanoparticle-titanium carbide (AuNPs-MXene) synergistic signal amplification, and electrochemical (EC) detection for precise quantification and visual dynamic monitoring of microRNA-181b (miR-181b) in osteosarcoma tissues. The platform utilizes microneedle arrays for minimally invasive tissue sample extraction and employs AuNPs-MXene composites to significantly enhance the sensing interface conductivity and biomolecule loading capacity. Experimental results show that the conductivity of the AuNPs-MXene composite (10.9 ± 1.1 S/cm) is nearly nine times higher than that of the Ti_3_AlC_2_ MAX phase (1.2 ± 0.12 S/cm). Under optimal conditions, the sensor achieves a linear detection range for miR-181b from 1 aM to 100 pM, with a detection limit as low as 0.34 aM, and exhibits favorable selectivity, high reproducibility and long-term stability of up to 60 days. The integrated visual interface enables real-time monitoring of the detection process and intuitive output of results. Validation using clinical tissue samples demonstrates that the platform can effectively distinguish between osteosarcoma patients and normal controls (p < 0.05), with an area under the receiver operating characteristic curve (AUC) of 0.889. This integrated platform combines minimally invasive sampling, efficient signal amplification, and visual detection, offering a novel point-of-care tool for dynamic monitoring of osteosarcoma.

## 1 Introduction

**SCHEME 1:**
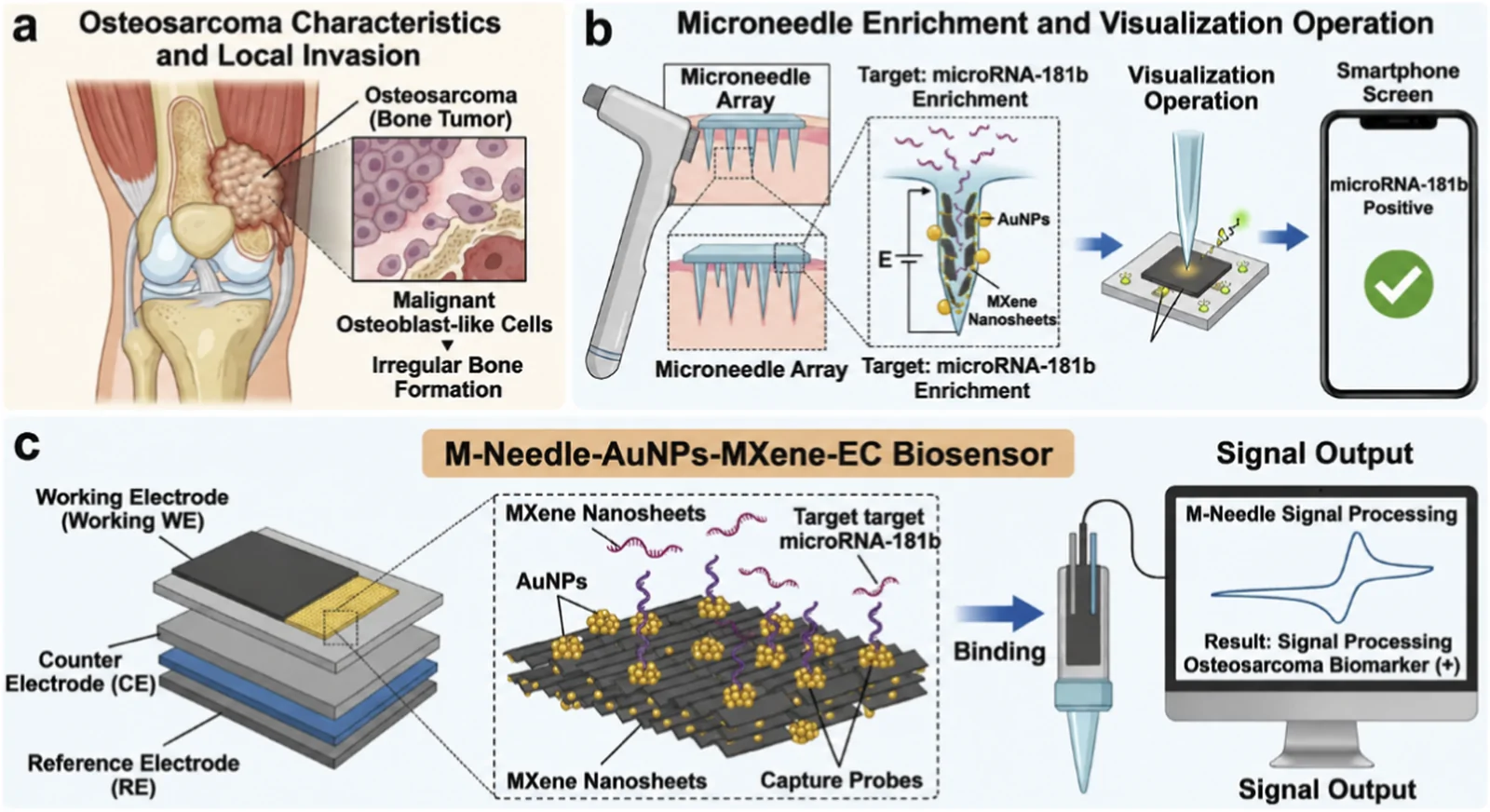
**(a)** Occurrence of osteosarcoma, **(b)** Microneedle and AuNPs-MXene materials enable detection sensitivity for microRNA-181b, **(c)** Detection principle of M-Needle-AuNPs-MXene-EC, where AuNPs enhance sensitivity and couple the dual platform, while MXene improves the mobility of the electrochemical sensor.

Osteosarcoma, the most common primary malignant bone tumor (Scheme 1a), predominantly affects children and adolescents. It is characterized by high malignancy, rapid progression, and a propensity for early lung metastasis, posing a serious threat to patient health (Jiang et al., 2022; Tian et al., 2023; Wang et al., 2024). Currently, the clinical management of osteosarcoma faces two major challenges: the lack of early warning methods for postoperative recurrence and metastasis, and the absence of dynamic and precise methods for evaluating therapeutic efficacy (Jiang et al., 2022; Tian et al., 2023; Wang et al., 2024). In this context, liquid biopsy techniques, particularly those targeting microRNAs, offer new avenues for precise management of osteosarcoma. Studies have shown that microRNA-181b (miR-181b) is aberrantly overexpressed in osteosarcoma tissues and participates in regulating tumor cell proliferation, invasion, and drug resistance by interacting with downstream target genes, making it a highly promising osteosarcoma-specific biomarker (Chen et al., 2023). However, current miRNA detection methods—predominantly qRT-PCR and microarrays—are poorly suited for point-of-care applications. Despite their sensitivity, these techniques entail cumbersome operation, bulky instrumentation, protracted turnaround, and high technical barriers (Aydemir et al., 2015; Nguyen et al., 2021; Zhang et al., 2025), thus failing to address the pressing need for portable, high-frequency monitoring of osteosarcoma.

Electrochemical biosensors demonstrate great promise for constructing portable biosensing platforms due to their rapid response, simple operation, ease of miniaturization, and low cost (Zahed et al., 2020; de Lima et al., 2021; Yang et al., 2021). To achieve highly sensitive detection of trace miRNAs in tissue samples, signal amplification strategies at the electrode interface are crucial. Transition metal carbides/nitrides (MXenes), as emerging two-dimensional nanomaterials, possess large specific surface areas, favorable conductivity, and abundant surface functional groups (Liu et al., 2025; Zheng et al., 2026), making them ideal electrode modification substrates that significantly enhance electron transfer rates and probe loading capacities (Hosseini et al., 2020; Yang et al., 2020). However, single-component MXene materials still have limitations in immobilizing biomolecules. Gold nanoparticles (AuNPs) not only inherit favorable conductivity but also enable efficient and stable coupling of thiol- or amine-modified biorecognition elements, such as nucleic acid aptamers, through gold-sulfur (Au-S) or gold-amine bonds (Narayanan et al., 2015). Therefore, the integration of AuNPs with MXene creates a synergistic sensing interface: MXene serves as a highly conductive three-dimensional scaffold, while AuNPs not only amplify electrochemical signals but also provide stable anchoring sites for thiolated aptamers via Au–S bonds. This dual-function architecture enables both high-sensitivity signal transduction and high-specificity target recognition for miR-181b.

Direct analysis of markers in osteosarcoma tissue samples provides a more accurate reflection of the local molecular pathological state compared to indirect samples such as blood. However, traditional tissue sample acquisition relies on biopsy, an invasive procedure that causes patient discomfort and hinders frequent dynamic monitoring. Microneedle technology offers an ideal solution to this challenge as a minimally invasive, low-pain method for sampling interstitial fluid or the tissue microenvironment (Ye et al., 2016; Yu et al., 2017; Kai et al., 2019). The flexible microneedle arrays employed in this study can efficiently penetrate tissue barriers and extract miR-181b-rich tissue fluid in a minimally invasive manner, providing direct samples for subsequent electrochemical detection. Furthermore, integrating the microneedle sampling unit with the electrochemical detection unit into a portable sensing interface not only simplifies the detection workflow and shortens analysis time but also enables high-frequency, point-of-care tracking of osteosarcoma patients at the bedside or even outside the hospital, providing real-time data support for personalized treatment and prognostic management.

Based on the above analysis, this study develops a highly sensitive and portable biosensing platform (M-Needle-AuNPs-MXene-EC, Scheme 1b,c) integrating flexible microneedle (M-Needle) sampling, gold nanoparticle-titanium carbide (AuNPs-MXene) synergistic signal amplification, and electrochemical (EC) detection for precise quantitative monitoring of miR-181b in osteosarcoma tissues. The platform utilizes microneedles for minimally invasive and efficient tissue sample extraction and leverages the favorable conductivity and high-affinity interface of AuNPs-MXene composites to achieve highly specific capture and significant electrochemical signal enhancement of the target miRNA, promising a novel point-of-care tool for postoperative recurrence warning, dynamic efficacy evaluation, and personalized diagnosis and treatment of osteosarcoma.

## 2 Experimental section

### 2.1 Synthesis of materials

#### 2.1.1 Synthesis of MXene (Ti_3_C_2_T_x_)

Two-dimensional Ti_3_C_2_T_x_ MXene was synthesized from the Ti_3_AlC_2_ MAX phase via a selective alkaline etching method. Specifically, 1.0 g of Ti_3_AlC_2_ powder (purity ≥98%) was added to 20 mL of 10 M sodium hydroxide (NaOH, purity ≥98%) aqueous solution (Xu et al., 2016; Eom et al., 2020; Iravani and Varma, 2021; Song et al., 2024). The mixture was vigorously stirred in a reactor at 180 °C for 24 h to selectively remove the aluminum layers. The resulting product was collected by centrifugation (3,500 rpm, 5 min). The precipitate was then washed with deionized water for a minimum of six cycles until the supernatant pH reached neutral (≤7) (Chen et al., 2018; Chen et al., 2021; Ge et al., 2024). Subsequently, the precipitate was redispersed in anhydrous ethanol and subjected to probe sonication for 30 min to exfoliate the multilayered particles, yielding a stable colloidal suspension of multilayered MXene.

#### 2.1.2 Synthesis of gold nanoparticles (AuNPs)

Gold nanoparticles were prepared using the sodium citrate reduction method. 100 mL of 0.01% chloroauric acid (HAuCl_4_) solution was heated to boiling. Under vigorous stirring, 1 mL of 1% sodium citrate solution was rapidly added (Sun et al., 2023; Shan et al., 2024; Hu et al., 2025). The solution color changed from pale yellow to wine red, after which heating and stirring were continued for 15 min. The solution was then naturally cooled to room temperature, yielding a colloidal AuNPs solution, which was stored at 4 °C for later use.

#### 2.1.3 Preparation of AuNPs-MXene composite

The AuNPs-MXene composite was prepared via a solution self-assembly method. 10 mL of MXene colloidal suspension (1 mg/mL) was mixed with 5 mL of AuNPs colloidal solution and magnetically stirred at room temperature for 2 h, followed by ultrasonication in a 60 °C water bath for 1 h to ensure uniform integration. The composite was separated by centrifugation (3,000 rpm, 5 min), washed three times with deionized water, and finally dried under vacuum overnight to obtain AuNPs-MXene composite powder.

### 2.2 Fabrication and characterization of flexible microneedle arrays

Flexible microneedle arrays were fabricated using a template replication method. Polydimethylsiloxane (PDMS) prepolymer and curing agent were mixed at a 10:1 ratio, poured onto a microneedle master mold, degassed under vacuum, and cured at 80 °C for 2 h. After curing, the mold was demolded to obtain the PDMS microneedle array. The morphology and structural integrity of the microneedles were characterized by scanning electron microscopy (SEM, FEI Nova NanoSEM 450, operating voltage 5 kV). Finite element analysis software (COMSOL Multiphysics) was used to simulate the stress distribution of the microneedle during tissue penetration.

### 2.3 Sensor fabrication and biofunctionalization

#### 2.3.1 Preparation of working electrode

Using the prepared flexible microneedle substrate and a mask plate with a designed structure, the M-Needle-AuNPs-MXene-EC electrode was fabricated. This was followed by ultrasonic cleaning in anhydrous ethanol and deionized water for 5 min each. Subsequently, 10 µL of the AuNPs-MXene composite dispersion was drop-coated onto the clean gold electrode surface and dried at room temperature to form the AuNPs-MXene modified electrode.

#### 2.3.2 Aptamer immobilization and blocking

To endow the sensor with specific recognition capability for miR-181b, 10 µL of thiolated miR-181b aptamer solution (sequence: 5′-SH-ACTCACCGACAGCGTTGAATGTT-3′) was dropped onto the AuNPs-MXene modified electrode surface and incubated overnight at 4 °C (Sasa et al., 2024). The electrode was then rinsed with 10 mM Tris-HCl buffer (pH 7.4) to remove unbound aptamers. Finally, the electrode was immersed in 1 mM 6-mercapto-1-hexanol (MCH) solution for 1 h at room temperature to block nonspecific adsorption sites, rinsed again with Tris-HCl buffer, and dried.

### 2.4 Material and device characterization

The morphology and microstructure of the synthesized materials and sensor interfaces were characterized using scanning electron microscopy (SEM, FEI Nova NanoSEM 450) and transmission electron microscopy (TEM, FEI Tecnai G2 F20). Elemental distribution was analyzed by energy dispersive X-ray spectroscopy (EDS). The conductivity of different materials (Ti_3_AlC_2_ MAX phase, MXene, AuNPs-MXene) was measured using a four-point probe resistivity tester (RTS-8). All electrochemical measurements were performed using an electrochemical workstation (CHI1040c) with a three-electrode system: the prepared modified electrode as the working electrode, an Ag/AgCl electrode as the reference electrode, and a platinum wire electrode as the counter electrode.

### 2.5 Electrochemical measurements and parameter optimization

Electrochemical measurements were conducted in 0.1 M KCl solution containing 10 mM [Fe(CN)_6_]^3-^/^4-^. Cyclic voltammetry (CV) was performed in the potential range of −0.2–0.6 V at a scan rate of 50 mV/s to characterize interface changes during electrode modification. The signal was measured using differential pulse voltammetry (DPV), with the scanning voltage set from −0.1 V to 0.3 V. The signal response was taken as the difference between the steady-state current and the background current. Key experimental parameters were optimized to achieve optimal detection performance: aptamer concentration (10–60 µM), incubation temperature (32 °C–46 °C), detection buffer pH (5.0–9.0), and incubation time (0–60 min).

### 2.6 Sensor performance evaluation

Under optimized experimental conditions, the performance of the constructed electrochemical sensor was systematically evaluated. Specificity was assessed by comparing the current responses of the sensor to 10 aM target miR-181b and 100 aM interferents (miR-21, miR-16, miR-146, and single-base mismatched sequence). Reproducibility was evaluated by calculating the relative standard deviation (RSD) of the response signals from 10 independently fabricated sensors detecting the same concentration (10 aM) of miR-181b. Stability was assessed by storing the fabricated sensors at 4 °C and measuring their response to the same concentration (10 aM) of miR-181b on days 1, 7, 14, 30, and 60. Sensitivity and dynamic range were determined by detecting different concentrations (1 aM, 10 aM, 100 aM, 1 fM, 10 fM, 100 fM, 1 pM, 10 pM, 100 pM) of miR-181b standards, fitting the current response against the logarithm of miR-181b concentration to obtain a standard curve, and calculating the limit of detection (LOD) using the 3σ method.

### 2.7 Clinical sample processing and detection

To validate the applicability of the sensor in real clinical scenarios, osteosarcoma tissue samples and adjacent normal tissue samples were processed and analyzed. Tissue samples were obtained from a collaborating hospital with ethics committee approval and uniformly cut to a size of 2 cm × 2 cm with a thickness of 5 µm. For RNA extraction, the flexible microneedle array was attached to the tissue surface, held under uniform pressure for 10 s, then removed and vortexed in RNA extraction buffer for 1 min to release RNA. For electrochemical detection, RNA sample was dropped onto the functionalized sensor surface, incubated at 37.5 °C for 30 min, rinsed with buffer, and the steady-state current was recorded using differential pulse voltammetry (DPV) under the same conditions as Section 2.5. The miR-181b concentration in the sample was calculated based on the standard curve, and each sample was measured in triplicate. Data acquisition and concentration calculation were performed using a custom Python script (version 3.9, Python Software Foundation). All human experimental protocols were approved by the Ethics Committee of Shanghai Sixth People’s Hospital (2022-YS-117).

### 2.8 Statistical analysis

All experimental data are presented as mean ± standard deviation (mean ± SD) and were statistically analyzed using GraphPad Prism 9.0 software. Differences between two groups were evaluated using unpaired two-tailed Student’s t-test. Diagnostic performance was analyzed using receiver operating characteristic (ROC) curves (Chauhan et al., 2020; Luo et al., 2024), and the area under the curve (AUC) was calculated.

## 3 Results

### 3.1 Fabrication and characterization of microneedle unit

**FIGURE 1:**
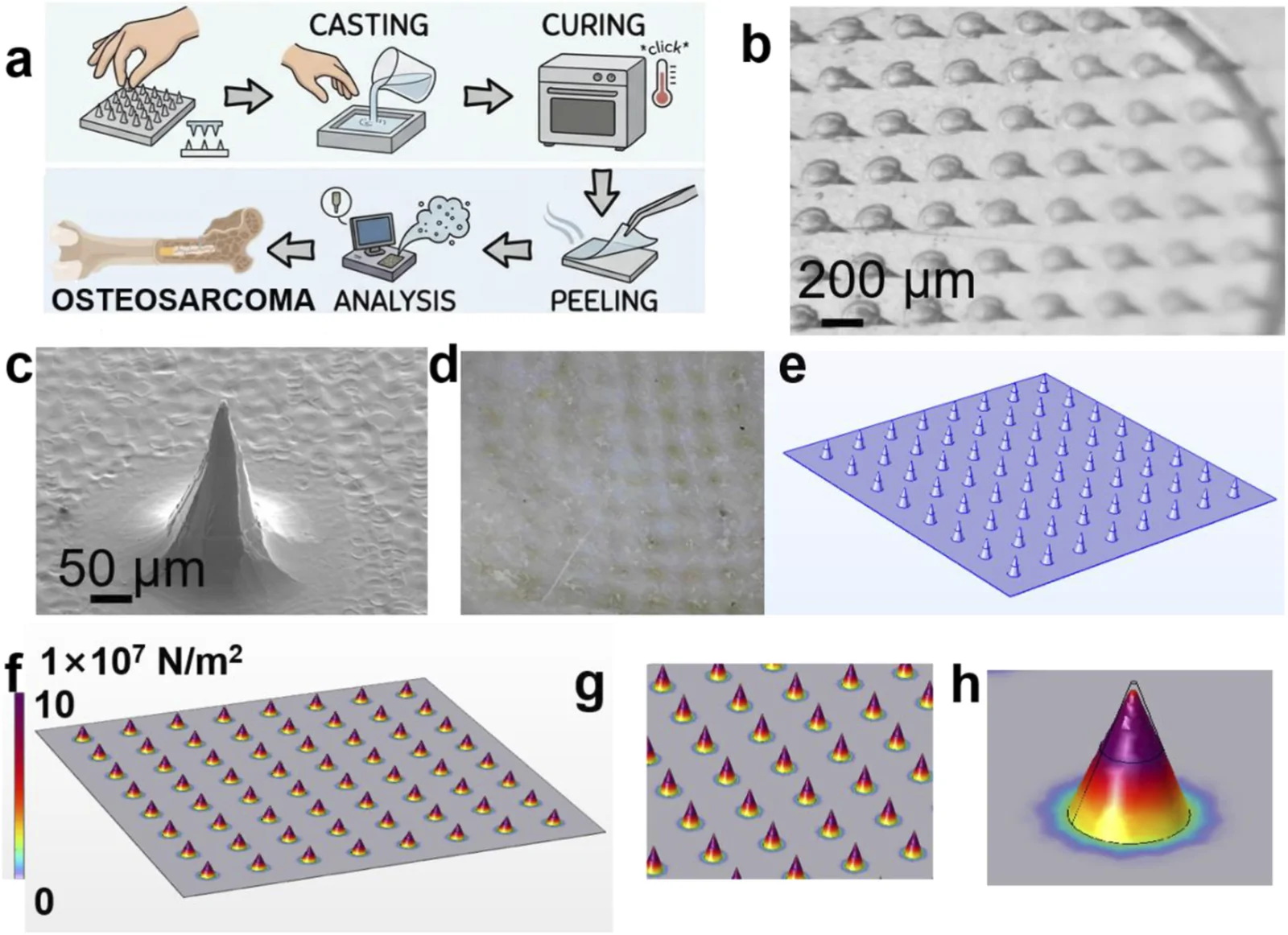
Design and characterization of the microneedle unit for the M-Needle-AuNPs-MXene-EC platform. **(a)** Schematic diagram of the fabrication process of flexible microneedles; **(b)** Optical image of the microneedle array; **(c)** Interface image of the microneedle array after tissue penetration; **(d)** Scanning electron microscopy (SEM) image of a microneedle, showing its sharp tip; **(e)** COMSOL schematic diagram of the microneedle; **(f–h)** simulated stress distribution of the microneedle, where the main stress is located at the needle base and middle regions, with a uniform distribution, which facilitates the extraction of microRNA-181b.

To enhance the efficiency of extracting microRNA-181b (miR-181b) from osteosarcoma tissue samples, we fabricated a flexible microneedle array (Figure 1a). The microneedles were prepared using a template replication method. After plasma treatment, they achieved efficient tissue penetration and miRNA enrichment. An optical image of the fabricated microneedle array is shown in Figure 1b, demonstrating a well-ordered and uniform array structure. To evaluate the tissue penetration capability of the microneedles, morphological analysis of the interface after tissue contact was performed (Figure 1c), revealing that the microneedles effectively penetrated the tissue barrier, forming a tight contact interface. Scanning electron microscopy (SEM) images further revealed the surface morphology of the microneedles (Figure 1d), showing sharp tips and intact structures. COMSOL simulations (Figures 1e–h) revealed that the principal stress concentrated at the needle base and middle shaft regions. This distribution pattern ensures mechanical integrity during insertion while promoting uniform tissue fluid permeation for efficient miR-181b extraction.

### 3.2 Structural characterization of AuNPs-MXene composite

**FIGURE 2:**
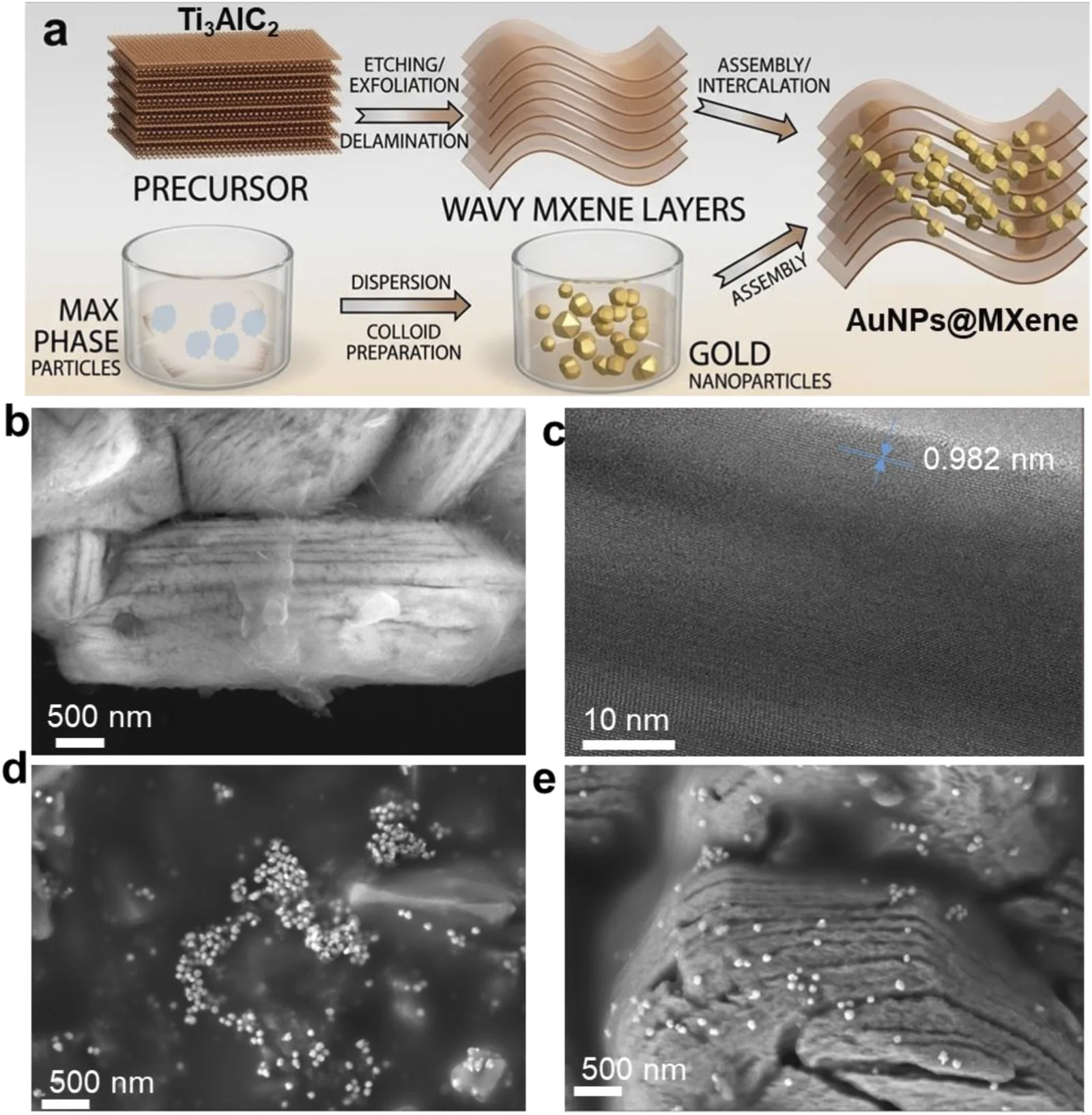
Synthesis and structural characterization of AuNPs-MXene composite. **(a)** Schematic diagram of the synthesis of AuNPs, MXene, and AuNPs-MXene composite; **(b)** SEM image of MXene, showing its typical layered structure; **(c)** TEM image of MXene, with a measured lattice spacing of 0.982 nm; **(d)** SEM image of AuNPs, with an average particle size of 88.09 ± 4.33 nm; **(e)** SEM image of the AuNPs-MXene composite, showing AuNPs uniformly attached to the MXene surface.

To achieve highly sensitive electrochemical detection of miR-181b, we constructed an AuNPs-MXene composite (Figure 2a). Scanning electron microscopy (SEM) images showed that the Ti_3_C_2_T_x_ MXene synthesized via the alkaline etching method exhibited a typical accordion-like layered structure (Figure 2b), which facilitated electrolyte ion diffusion and provided abundant electrochemical active sites. Transmission electron microscopy (TEM) further revealed the layered details of MXene, with an interlayer spacing of approximately 0.982 nm (Figure 2c), consistent with literature reports (Wu et al., 2023). Gold nanoparticles (AuNPs) exhibited a uniform spherical morphology under scanning electron microscopy (SEM, Figure 2d). Statistical analysis of 36 randomly selected nanoparticles from the SEM images revealed an average diameter of 88.09 ± 4.33 nm (Supplementary Figure S1), confirming the good uniformity and narrow size distribution of the synthesized AuNPs. After hydrothermal assembly of AuNPs with MXene, SEM images showed that AuNPs were uniformly attached to the layered surface of MXene (Figure 2e), forming a close heterointerface. This structure preserved the high conductivity of MXene while providing a favorable platform for efficient and stable coupling of nucleic acid aptamers.

### 3.3 Optimization of the M-needle-AuNPs-MXene-EC sensing interface

**FIGURE 3:**
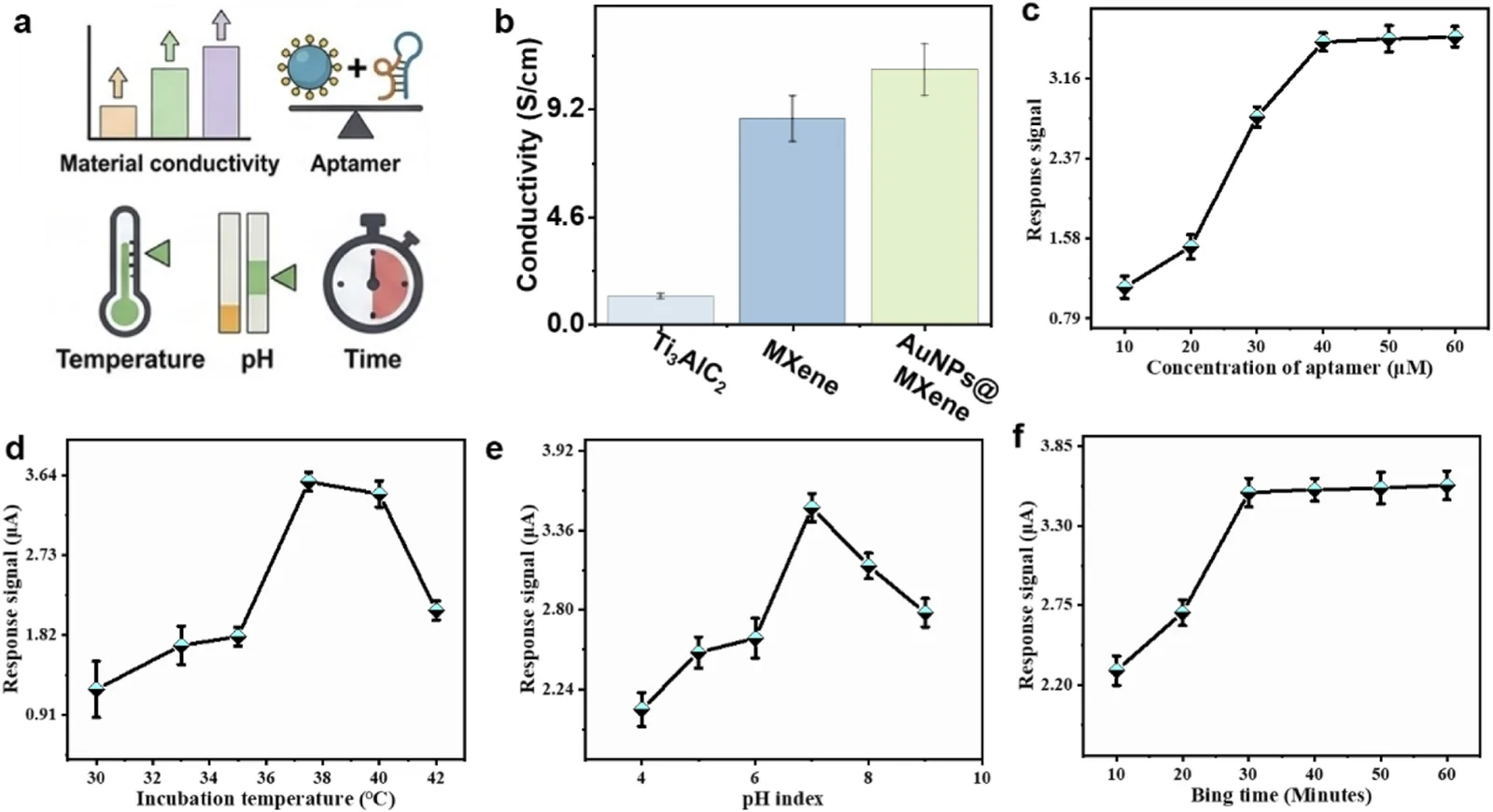
Performance optimization of the M-Needle-AuNPs-MXene-EC sensor. **(a)** Schematic diagram of the parameters involved in sensor optimization; **(b)** Comparison of conductivity of Ti_3_AlC_2_, MXene, and AuNPs-MXene composite (n = 3); **(c)** Effect of aptamer concentration on sensor response, with an optimal concentration of 40 μM; **(d)** Effect of incubation temperature on sensor response, with an optimal temperature of 37.5 °C; **(e)** Effect of pH on sensor response, with an optimal pH of 7.0; **(f)** Binding kinetics curve, with signal saturation reached at 30 min.

Key parameters affecting the performance of the M-Needle-AuNPs-MXene-EC sensor were systematically optimized (Figure 3a). First, the effect of different material modifications on conductivity was evaluated using the four-point probe method (Figure 3b). The pristine Ti_3_AlC_2_ MAX phase exhibited a conductivity of 1.2 ± 0.12 S/cm. After etching to remove the Al layer, the conductivity of MXene increased to 8.2 ± 0.98 S/cm. After further introducing AuNPs, the conductivity of the AuNPs-MXene composite significantly increased to 10.9 ± 1.1 S/cm, demonstrating the synergistic effect of MXene and AuNPs in enhancing electron transport.

To optimize the biorecognition interface, four key parameters—aptamer concentration, incubation temperature, buffer pH, and incubation time—were systematically investigated (Figures 3c–f). For aptamer concentration (Figure 3c), the signal intensity increased from 10 μM to 40 μM but decreased at higher concentrations up to 60 μM, indicating 40 μM as the optimal loading for maximal probe immobilization and target accessibility. Incubation temperature optimization (Figure 3d) showed that the response reached a maximum at 37.5 °C within the tested range of 30 °C–42 °C, consistent with the thermodynamics of nucleic acid aptamer–target hybridization. pH optimization (Figure 3e) revealed that the sensor exhibited the strongest signal at neutral pH 7.0, which favours maintaining aptamer conformation and interfacial stability. Finally, binding kinetic analysis (Figure 3f) demonstrated that the signal reached saturation after approximately 30 min of incubation; therefore, 30 min was selected as the optimal incubation time for subsequent experiments.

### 3.4 Analytical performance evaluation of the M-needle-AuNPs-MXene-EC sensor

**FIGURE 4:**
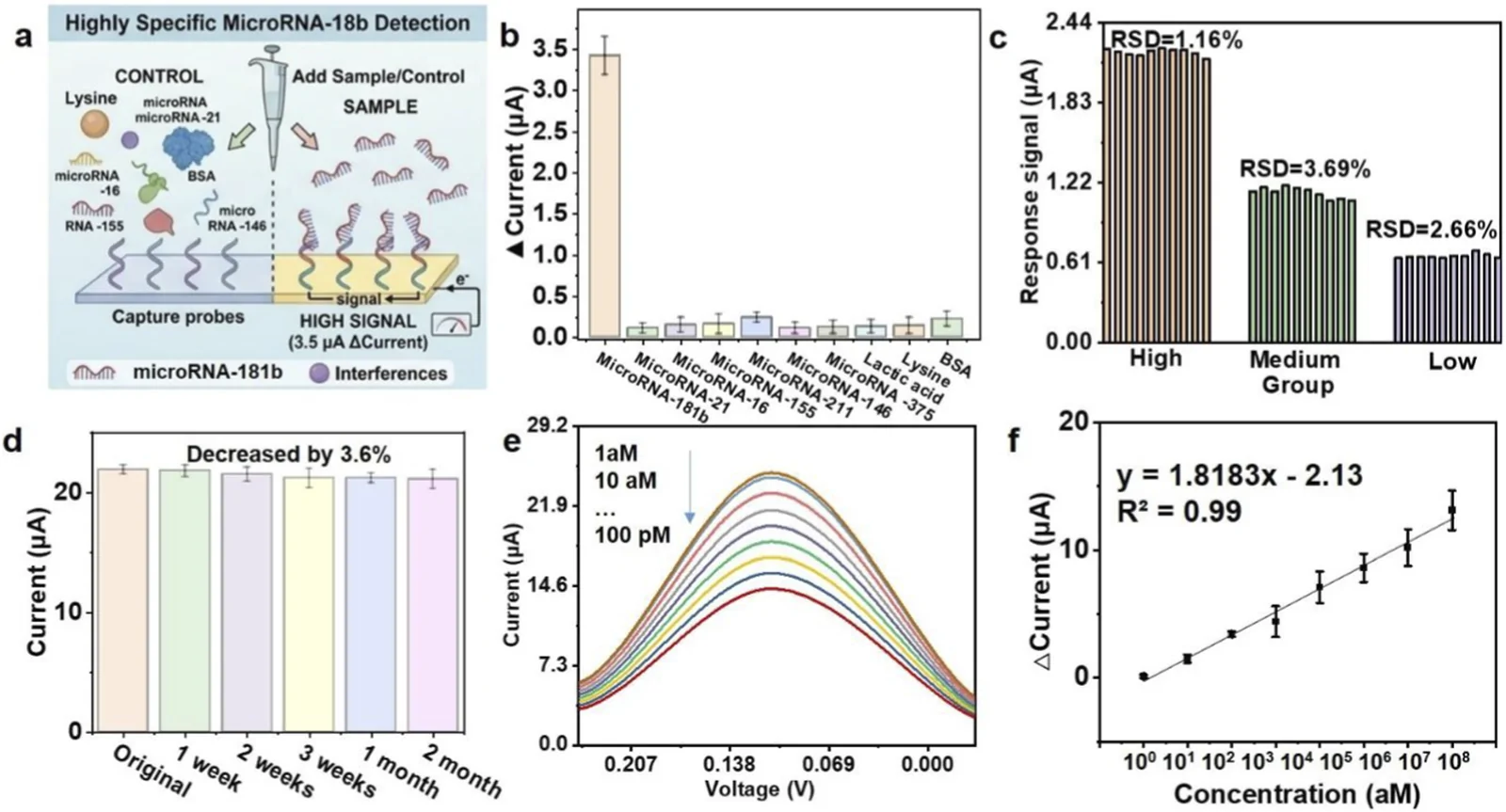
Performance evaluation of the M-Needle-AuNPs-MXene-EC sensor. **(a)** Schematic diagram of the sensor specificity detection principle; **(b)** Comparison of sensor responses to miR-181b and various interferents (miR-21, miR-16, miR-146, lactate, miR-375, etc.), demonstrating favorable selectivity; **(c)** Batch-to-batch reproducibility of 30 sensors for three different concentrations; **(d)** Long-term stability evaluation of the sensor over 2 months, with signal decay of 3.61%; **(e)** Response curves of the sensor detecting different concentrations of miR-181b (1 aM–100 pM); **(f)** Standard curve based on the response signals (y = 1.8183x - 2.13, *R* ^2^ = 0.99).

Under optimized conditions, the analytical performance of the M-Needle-AuNPs-MXene-EC sensor was systematically evaluated (Figure 4a). Specificity experiments (Figure 4b) demonstrated that the current response of the sensor to the target miR-181b (10 aM) was much higher than that to non-complementary miRNAs (including miR-21, miR-16, miR-146) and common biological interferents. To further evaluate potential interference from other types of analytes, we also tested lactate (a metabolic biomarker) and microRNA-375 (another osteosarcoma-related miRNA), both of which produced only negligible current responses, confirming that the M-Needle-AuNPs-MXene-EC platform exhibits favorable specificity even in the presence of structurally and chemically diverse interferents. To evaluate the batch-to-batch reproducibility of the M-Needle-AuNPs-MXene-EC sensor for detecting samples at different concentrations (Figure 4c), we set up three groups corresponding to high, medium, and low concentrations. Using 30 sensors in total (10 sensors per group), the relative standard deviations (RSDs) for the three groups were 1.16%, 3.69%, and 2.66%, respectively. These results indicate that the M-Needle-AuNPs-MXene-EC sensor exhibits good reproducibility in signal response to high, medium, and low concentrations when handling a large number of samples. Long-term stability experiments (Figure 4d) showed that after storage at 4 °C for 2 months, the initial signal of the sensor only decayed by 3.61%, demonstrating favorable stability.

Sensor sensitivity was evaluated by detecting different concentrations of synthetic miR-181b standards (Figure 4e). As the miR-181b concentration increased from 1 aM to 100 pM, the electrochemical signal response increased. Linear fitting of the signal response against the logarithm of concentration (Figure 4f) yielded a standard curve equation of y = 1.8183x - 2.13, with a correlation coefficient *R* ^2^ = 0.99. Based on three times the standard deviation of the blank signal, the LOD was determined to be 0.34 aM (de Lima et al., 2021; Wu et al., 2024). This sensitivity is approximately two orders of magnitude lower than typical qRT-PCR detection limits, demonstrating the platform’s suitability for trace-level miRNA analysis in clinical specimens.

### 3.5 Visual interface of the M-needle-AuNPs-MXene-EC platform

**FIGURE 5:**
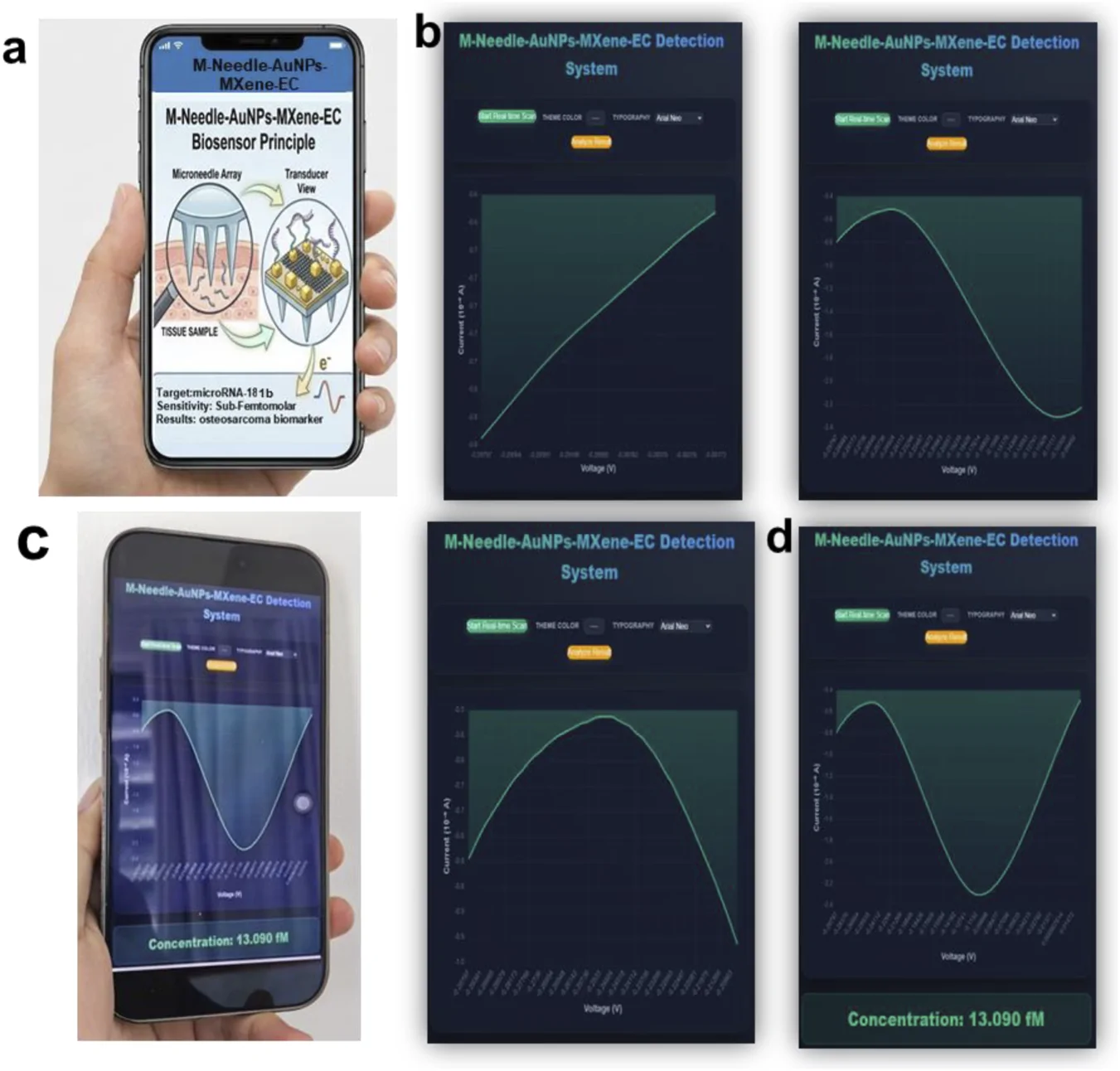
Visual interface of the M-Needle-AuNPs-MXene-EC platform. **(a)** Schematic diagram of the visual interface design; **(b)** Interface screenshot at the start of detection; **(c)** Photograph of the visual interface; **(d)** Direct output of miR-181b concentration after detecting a clinical sample using this interface.

To achieve portability and intuitive visualization, a visual interface was designed and integrated into the M-Needle-AuNPs-MXene-EC platform (Figure 5a). This interface enabled real-time display of dynamic signal changes during the detection process. Figure 5b shows the interface status at the start of detection, while Figure 5c shows a photograph of the visualization system, demonstrating its simple interface and convenient operation. Using this platform to detect a clinical tissue sample, the interface automatically calculated and directly output the miR-181b concentration in the sample as 13.09 fM (Figure 5d) using the built-in algorithm and standard curve (Supplementary Figure S2). The data processing algorithm was implemented in Python (version 3.9), ensuring rapid and user-friendly readout for point-of-care applications. This result validated the practicality of this integrated visual interface for point-of-care testing of clinical samples.

### 3.6 Clinical sample validation and diagnostic performance evaluation

**FIGURE 6:**
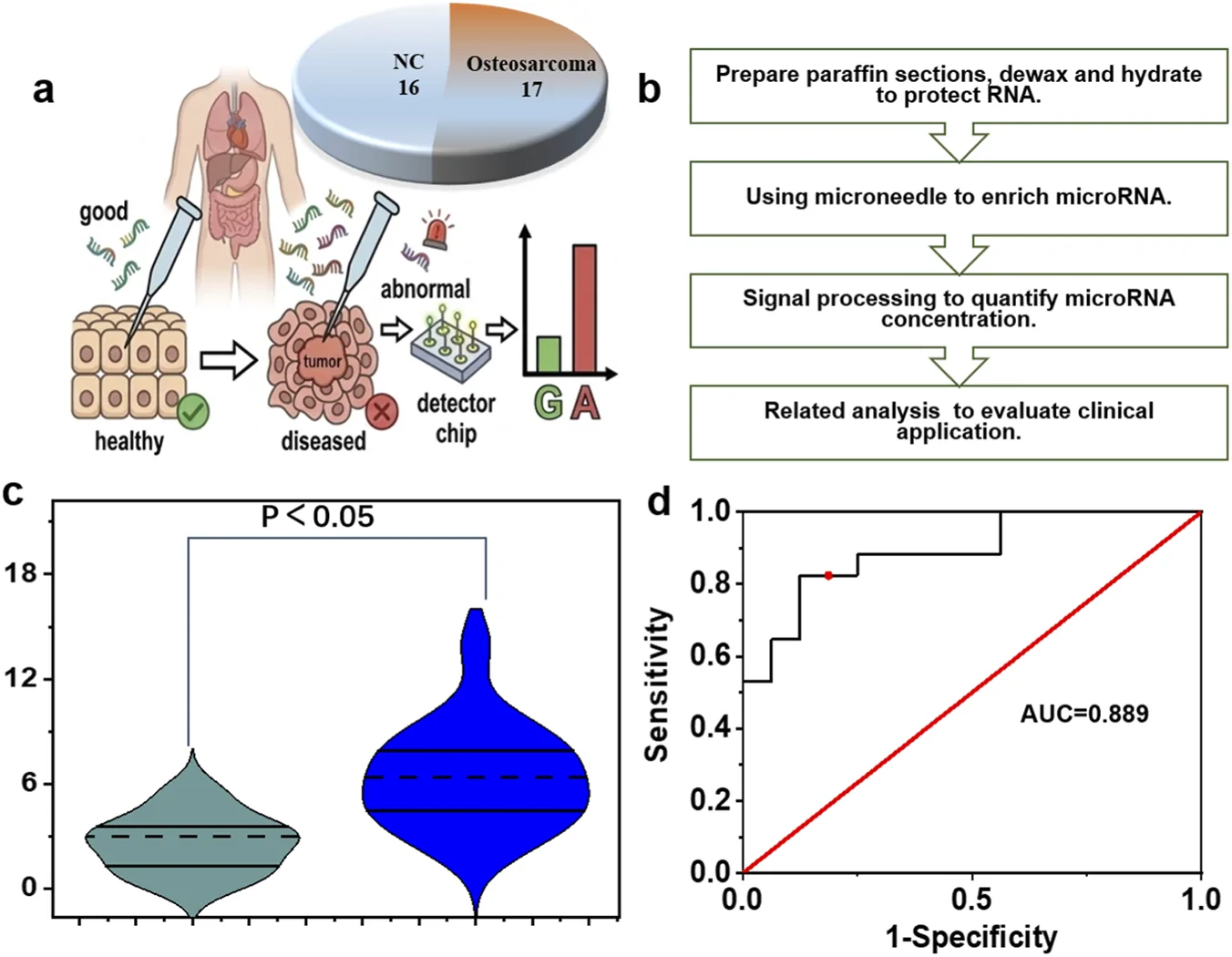
Clinical validation and diagnostic performance evaluation of the M-Needle-AuNPs-MXene-EC platform. **(a)** Schematic diagram of the clinical cohort, including 17 osteosarcoma and 16 normal control tissue samples; **(b)** Microneedle-based clinical sample detection workflow; **(c)** Statistical analysis of miR-181b levels detected by the sensor in the two sample groups, p < 0.05, indicating a highly significant difference; **(d)** ROC curve analysis for evaluating diagnostic performance, AUC = 0.889.

To evaluate the clinical applicability of the M-Needle-AuNPs-MXene-EC platform, we analyzed 33 osteosarcoma tissue samples (17 osteosarcoma, 16 normal controls) (Figure 6a). The entire detection workflow included tissue sample processing, microneedle-assisted RNA extraction, sensor detection, and data analysis (Figure 6b). Figure 6c shows the sensor detection results for the two sample groups. Statistical analysis revealed a highly significant difference in miR-181b levels between the osteosarcoma group and the normal control group (p < 0.05), indicating that the platform could effectively distinguish osteosarcoma from normal tissue samples. Further evaluation of diagnostic performance using receiver operating characteristic (ROC) curve analysis (Figure 6d) showed an area under the curve (AUC) of 0.889, indicating high diagnostic accuracy. These clinical data fully validated the potential of the M-Needle-AuNPs-MXene-EC platform for quantifying miR-181b in osteosarcoma tissue samples, providing a powerful tool for clinical monitoring of osteosarcoma.

## 4 Discussion

### 4.1 Synergistic advantages of MXene and AuNPs in electrochemical sensing

Two-dimensional transition metal carbides/nitrides (MXenes) have shown great promise for constructing high-performance electrochemical biosensors due to their high conductivity, large specific surface area, and good biocompatibility. In this study, we selected Ti_3_C_2_T_x_ MXene as the electrode modification substrate. Its unique accordion-like layered structure (Figures 2b,c) provided fast channels for electron transport and an ideal platform for high-density immobilization of biomolecules. SEM and TEM characterization (Figure 2) confirmed the layered morphology of MXene and the successful loading of AuNPs. Conductivity measurements (Figure 3b) showed that the conductivity of the AuNPs-MXene composite (10.9 ± 1.1 S/cm) was significantly higher than that of pristine MXene (8.2 ± 0.98 S/cm) and the MAX phase precursor (1.2 ± 0.12 S/cm). This enhancement could be attributed to the synergistic effect between AuNPs and MXene: highly conductive AuNPs acted as “conductive bridges” between MXene nanosheets, effectively reducing the inter-sheet contact resistance and constructing an efficient three-dimensional conductive network. Simultaneously, AuNPs provided abundant gold-sulfur (Au-S) binding sites for efficient and stable coupling of the miR-181b aptamer, ensuring high specificity and affinity of the sensing interface. Despite the demonstrated synergistic enhancement, the underlying interfacial electron transfer mechanism between AuNPs and MXene requires further systematic investigation. Future work should combine *in situ* characterization and theoretical calculations to optimize the composite ratio and spatial arrangement for maximum sensing performance.

### 4.2 Clinical translation value of microneedle-assisted sampling

Traditional tissue biopsy for molecular analysis suffers from limitations such as invasiveness, sampling difficulty, and inability to perform dynamic monitoring. The flexible microneedle array employed in this study (Figure 1) offered an effective solution to this clinical bottleneck. Microneedles could minimally invasively penetrate tissue barriers to efficiently extract trace tissue RNA (Figures 1c–f), maintaining structural integrity after puncture (Figure 1f), ensuring sampling reproducibility and stability. Integrating the microneedle sampling unit with the electrochemical detection unit into the M-Needle-AuNPs-MXene-EC platform achieved seamless integration from sample acquisition to signal readout. This integrated design not only simplified operational procedures and shortened analysis time but, more importantly, enabled high-frequency, dynamic molecular monitoring of osteosarcoma patients at the bedside. This holds significant clinical importance for early warning of postoperative recurrence and real-time assessment of chemotherapy efficacy. Nevertheless, we acknowledge that the current platform still has certain limitations for true point-of-care testing (POCT), such as the requirement for external electrochemical workstations and manual operation steps, which may hinder its use in resource-limited or bedside settings; future efforts will focus on integrating miniaturized potentiostat modules and automating the sampling-to-readout workflow to address these challenges. Although clinical testing yielded a promising AUC of 0.889, this preliminary validation was constrained by a modest sample cohort from a single institution. Future multicenter studies with larger populations and standardized protocols are essential to confirm diagnostic reliability and facilitate clinical adoption.

### 4.3 Application potential of the M-needle-AuNPs-MXene-EC platform in osteosarcoma diagnosis

Precise quantification of miR-181b levels in osteosarcoma tissues is crucial for assessing tumor progression, predicting prognosis, and monitoring treatment response. The M-Needle-AuNPs-MXene-EC platform developed in this study demonstrated significant advantages in analytical performance. Its ultra-wide dynamic range (1 aM–100 pM) and ultra-low detection limit (0.34 aM) could cover the physiological concentration range of miRNAs in clinical samples, with sensitivity far exceeding that of traditional qRT-PCR methods. More importantly, direct sampling from tissues using microneedles provided a more accurate reflection of the local molecular pathological state compared to indirect samples such as blood. Clinical sample validation results (Figures 6c,d) showed that the platform could distinguish osteosarcoma from normal tissue with high statistical significance and good diagnostic accuracy (AUC = 0.889). This performance advantage stemmed from the synergistic combination of microneedle sampling, AuNPs-MXene signal amplification, and electrochemical detection. Beyond tissue analysis, future work will also explore the feasibility of adapting this platform for minimally invasive detection of circulating miR-181b in peripheral blood or urine, which would further enhance its utility for non-invasive, dynamic monitoring of osteosarcoma progression and treatment response.

## 5 Conclusion

In this study, we successfully developed an M-Needle-AuNPs-MXene-EC biosensing platform integrating flexible microneedle sampling, AuNPs-MXene signal amplification, and electrochemical detection for highly sensitive and visual monitoring of miR-181b in osteosarcoma tissues. By combining AuNPs with MXene, the conductivity of the material was increased to 10.9 ± 1.1 S/cm, laying the foundation for constructing a high-performance sensing interface. The sensor achieved a detection limit as low as 0.34 aM for miR-181b, with a dynamic range spanning eight orders of magnitude, and exhibited favorable specificity, high reproducibility, and stability over 60 days. The integrated visual interface enabled intuitive monitoring of the detection process and real-time output of results, simplifying operational procedures. In validation using clinical osteosarcoma tissue samples, the platform effectively distinguished tumor from normal tissue (p < 0.05), with a diagnostic accuracy (AUC) of 0.889. This platform combines minimally invasive sampling, highly sensitive detection, and a visual interface, offering a highly promising point-of-care tool for portable monitoring of osteosarcoma. Nevertheless, the current configuration still relies on external electrochemical workstations and manual sample handling steps. Future iterations will focus on fully integrated, automated device design to enable true bedside operation in resource-limited settings.

## Data Availability

The original contributions presented in the study are included in the article/Supplementary Material, further inquiries can be directed to the corresponding authors.
