# Supplementary material for: Flexible microneedle array with AuNPs/MXene for visualized electrochemical monitoring of osteosarcoma miR-181b
Source: Front Bioeng Biotechnol. 2026 Aug 10;14:1918744. doi: 10.3389/fbioe.2026.1918744 (PMC13500536; doi:10.3389/fbioe.2026.1918744)
Supplement: Supplementary file 1 [file Supplementaryfile1.docx]

**Flexible microneedle array with AuNPs/MXene for visualized electrochemical monitoring of osteosarcoma miR-181b**

Zhong-Sheng Zhu^#^, Hui Zhang^#^, Changchun Lu^#^, Li Du, Shuai Lu, Xin-Qi Cao, Ai Zhong Ding^*^, Nuo Yin^*^

Shanghai University of Medicine & Health Science Affiliated Sixth People’s Hospital South Campus, Shanghai, 201499, P.R. China.

#Contributed equally to this work

*Correspondence: Ai Zhong Ding, Email: [dz960809@163.com](mailto:dz960809@163.com); Nuo Yin, Email: [yn0102067@126.com](mailto:yn0102067@126.com)

**Supplementary Figure S1.** Statistical analysis of the particle size of the synthesized gold nanoparticles was performed based on SEM images.

**Supplementary Figure S2.** Detection stage for M-Needle-AuNPS-MXene-EC.
